# Recommendations for the Development of a Mobile HIV Prevention Intervention for Men Who Have Sex With Men and Hijras in Mumbai: Qualitative Study

**DOI:** 10.2196/publichealth.9088

**Published:** 2018-05-03

**Authors:** Shruta Rawat, J Michael Wilkerson, Sylvia M Lawler, Pallav Patankar, BR Simon Rosser, Kanjani Shukla, Seyram Butame, Maria L Ekstrand

**Affiliations:** ^1^ The Humsafar Trust Mumbai India; ^2^ School of Public Health University of Texas Health Science Center at Houston Houston, TX United States; ^3^ School of Public Health University of Minnesota Minneapolis, MN United States; ^4^ Minnesota Department of Health Minneapolis, MN United States; ^5^ University of California San Francisco Center for AIDS Prevention Studies San Francisco, CA United States

**Keywords:** health promotion, health seeking, gays and lesbians, Internet, HIV/AIDS

## Abstract

**Background:**

As Internet and mobile phone use expands in India, there is an opportunity to develop mobile health (mHealth) interventions for marginalized populations, including men who have sex with men (MSM) and hijras (transgender women), hesitant to access traditional health care systems.

**Objective:**

The purpose of this study was to determine if an mHealth intervention was acceptable to MSM and hijras living in Mumbai, and if so, what features would be useful in targeting the prevention of HIV acquisition and to increase the quality of life among persons living with HIV/AIDS.

**Methods:**

Data from 4 focus groups with MSM and interviews with 4 hijras, 10 health service providers, and 8 mHealth developers were thematically analyzed.

**Results:**

Once the need for an mHealth intervention was confirmed, comments about features were organized into 3 themes: content, interface, and retention. Content subthemes included providing sex education for younger community members, providing information about STIs, and providing information and social support for persons living with HIV. Interface subthemes included presenting content using pictures; using videos to present stories of role models; using push notifications for testing, appointment, and medication reminders; using geolocation to link to just-in-time services; and using telemedicine to increase access to health service providers and community services. The 5 retention subthemes included keeping it fun, using gaming mechanics, developing content in regional languages, protecting confidentiality, and linking to social networking apps.

**Conclusions:**

These findings may help inform mHealth development in India.

## Introduction

The National AIDS Control Organization estimates the prevalence of HIV among adults in India as 0.3% [1]. Among men who have sex with men (MSM) and hijras (transgender women), the prevalence of HIV is estimated to be 4.3% and 7.5%, respectively [1,2]. These relatively high rates of HIV among MSM and hijras have been attributed to individual-level risk behaviors like high rates of sexual concurrency (wherein individuals have multiple overlapping sexual partners) and low rates of condom use [3,4]. Additionally, MSM and hijras often face stigma, discrimination, and, in some cases, criminalization [5-7]. Due to fear of social recrimination, MSM and hijras may be unwilling to engage with health service providers, leaving them with unmet health care needs [7]. Some evidence suggests interventions using mobile health (mHealth) technology may increase access to health care in developing countries such as India [8]. Therefore, Indian MSM and hijras may also benefit from mHealth interventions.

In developing countries, including India, mHealth interventions have historically made use of short message service (SMS) technology [9-14]. However, the mobile technology market in India is complicated by governmental regulations, including a National Customer Preference Register, that limit the number of SMS messages sent and received per day [15]. Due to these limitations, researchers investigating the effects of SMS mHealth interventions in India have obtained mixed results [16,17]. It is estimated that in 2018 there will be 530 million mobile phone users in India, nearly twice that of the United States [18]. Given the challenges of harnessing SMS messaging for health interventions, researchers instead should consider the burgeoning mobile phone market within India as a means of advancing the field of mHealth, particularly in support of the unique health needs of MSM and hijras [19]. The difficulties in harnessing SMS technology for mHealth interventions make mobile phone technology such as Internet-enabled apps a viable option for public health interventionists.

In Western countries, many mHealth interventions capitalize on mobile phones to deliver HIV prevention messaging and needs assessments for high-risk MSM. Preferred content often includes information about HIV and sexually transmitted infections (STIs), proper condom use, behavioral risk reduction strategies, and partner communication strategies [20,21]. For persons living with HIV/AIDS (PLHA), preferred content includes information about health care providers and treatment, especially regarding linkage and retention into care and medication adherence [22,23]. Additional features include use of geolocation of HIV testing centers; automated reminders for testing, appointment, and medication adherence; use of existing public or private social networking apps and chat rooms to increase social support; links to role model videos; self-assessment tools; access to sexual health experts; referrals to health service providers; and telemedicine [22,24-27].

The successes and innovations of mobile phone–based mHealth interventions in Western populations make a strong case for developing such interventions for MSM and hijras in India. Thus, in this paper we report qualitative findings from a formative research study with high-risk Indian MSM and hijras, health service providers with MSM and hijra clientele, and mHealth developers to determine what content and features they would like to be included in an mHealth intervention to prevent new HIV infections and increase the quality of life among PLHA. To the best of our knowledge, there is no information about the preferred features of an mHealth intervention targeting high-risk MSM and hijras in India.

## Methods

### Study Population and Recruitment

We used a convenience sample to recruit study participants based on key informants identified by the research team and The Humsafar Trust, one of India’s largest nongovernmental organizations that advocates for the rights of sexual and gender minority people. We used snowball sampling [22,25] to recruit MSM into focus groups. Approximately 6 individuals were in each focus group. Hijras and health service providers were asked to participate in individual interviews. Eligibility criteria for MSM and hijras included being 18 years or older, living in Mumbai, and having used Internet-enabled technology (ie, laptop, desktop, tablet, or mobile phone) to meet male sex partners [28]. Health service providers included medical doctors, nurses, mental health providers, and outreach workers who had a significant number of MSM and hijra clients and who felt confident in their ability to speak about using technology to interact with patients. mHealth developers were recruited from an mHealth conference listserv. Conference organizers allowed the research team access to the email addresses of all attendees. Developers who had experience working with Indian populations were asked to contact the research team. Upon completion of focus group or interview, participants received Rs 300 (US $7). All study procedures were approved by the institutional review boards of all authors’ home institutions and the Indian Government.

The median age of the 24 MSM who participated in focus groups was 27 (interquartile range, IQR, 24 to 33) years. Most of them (21/24, 88%) were college educated. Nearly all the MSM (23/24, 99%) identified as gay or homosexual. About a third of them (9/24, 38%) were out about their sexual or gender identity to most or all the people they knew, 21% (5/24) were out to about half the people they knew, and 42% (10/24) of MSM participants were out to only a few people or no one at all.

The median age of the 4 hijras who participated in individual interviews was 25 (IQR 22 to 29) years. Three were college educated and all identified as hijras. Three were out to most of the people they knew as a hijra, while 1 was out to about half the people she knew.

The median age of the 10 health service providers who participated in individual interviews was 31 (IQR 28 to 38) years. Half of them (5/10, 50%) were college educated; 8 of the health service providers identified as men, 1 as a woman, and 1 preferred not to identify. On average, the health service providers had worked in prevention for 6.2 (SD 5.4) years and with MSM or hijras specifically for 5.3 (SD 4.9) years.

The median age of the 8 mHealth developers who participated in individual interviews was 39 (IQR 32 to 44) years, and all were college-educated men. On average, the mHealth developers had worked in mHealth for 4.0 (SD 2.9) years and had worked in India on average for 3.3 (SD 2.9) years. All the developer participants believed that mHealth interventions could be useful, although they acknowledged that developing such a tool for the Indian context would be challenging due to differences in languages spoken, literacy, and access to technology.

### Study Procedures

Prior to data collection, a member of the research team reviewed consent forms with all participants. After consenting but prior to the start of the qualitative data collection, participants completed a brief demographic questionnaire. Four focus groups [29] were conducted with MSM who used technology to meet male sex partners. Because it was difficult to identify hijras who use technology to meet sex partners—perhaps due to differences in income between MSM and hijras—the few who were identified were interviewed individually. Topics discussed in focus groups and interviews were similar. Both began with a discussion about participants’ experiences using technology to meet sex partners and concluded with a discussion about suggestions for an HIV/STI prevention mHealth intervention. Additionally, we interviewed 10 health service providers who had experience working with MSM and hijra patients. The interviews focused on the ways health service providers and their organizations might use technology-based interventions to address the health needs of MSM and hijras. Each focus group lasted approximately 1.5 hours, and each individual interview lasted approximately 1 hour. Although focus groups and interviews were conducted in the preferred language of participants (Marathi, Hindi, or English), most focus groups and interviews occurred in English. During the focus groups, participants often switched between English and either Hindi or Marathi. Copies of the semistructured focus group and interview protocols are available upon request from the corresponding author.

### Data Analysis

All focus groups and interviews were audiorecorded and transcribed. When participants spoke Hindi or Marathi, the recordings were translated into English by staff at The Humsafar Trust. The first and third authors, who are fluent in Marathi, Hindi, and English, compared the translated transcripts for accuracy. Because many focus group and interview participants spoke in English at least some of the time, the words of participants were retained for analysis. The English portions of the transcripts were not altered to adhere to English grammar rules. The English transcripts were entered into NVivo 10 (QSR International Pty Ltd) for content analysis [30]. Independently, the first and second authors coded all transcripts for distinct themes based on the meaning of words or phrases and then came together to compare codes and arrive at a common coding taxonomy by examining codes for frequency, strength, and relationship. The taxonomy was presented to and vetted by the entire research team to arrive at a final taxonomy, which was used to code all transcripts.

## Results

Three overarching themes emerged from the data that were relevant to the development of an mHealth HIV prevention intervention for Indian MSM: content, interface, and retention features of the intervention.

### Content

Content recommendations for an mHealth intervention included providing sex education for youth, providing information about STIs, and providing information and social support for PLHA. Because sex education is not uniformly taught in schools, participants suggested mHealth could provide missing sex education. Topics such as gender, identity, and sexuality as well as HIV/STI facts, proper condom use, and condom use negotiation skills were identified as being the most salient.

The way sex should be done, condom should be used and it should be done at safe place, all this should be shown.

Hijra

Participants also expressed a need for an intervention to address reasons why younger MSM and hijras might engage in condomless anal sex (barebacking), including possible coercion by older partners and perceived HIV status of a potential sex partner based on their appearance.

When I was 17, people used to persuade me to have bareback sex. Thankfully, I was aware of not having sex but they used to say, “Do it, nothing [bad] would happen.” So, the young guys would want to try out everything so that they might just fall for it.

MSM

If you smell good, wear good perfume, have nice underwear, and if you are physically clean then you are considered to be [HIV negative].

MSM

Participants viewed an mHealth intervention as a tool to provide accurate information on STIs. MSM and hijra participants recommended that the intervention have adequate information on STIs to aid in the identification of STIs prior to having sex with a potential partner*.* The health service providers shared this view and acknowledged that an mHealth intervention could also bridge gaps in understanding that health service providers have about STIs. Health service providers expected an mHealth intervention to promote a fear-based approach to STIs with graphic pictures of infections to scare the community members into practicing safer sex; however, MSM and hijras expressed an aversion to this approach. Thus, while there was agreement about the need for information about STIs, health service providers and MSM and hijras had divergent views on how best to deliver the information.

MSM, hijras, and health service providers viewed an mHealth intervention as a potential source of information and support for PLHA. Topics included ways to improve positive living, including medication adherence, how to contact a health care worker in cases of emergency hospitalizations, information about health centers offering various services for PLHA, and an online support group for respondents. Participants suggested that an mHealth intervention could connect individuals with health care workers who could provide support to PLHA in case of emergency as well as routine hospitalizations and with other persons living with the virus.

Sometimes PLHA and MSM community people [are] hospitalized and they don’t have their family members with them. At that time, there is a major problem with their care... so if we can do it by app...we can get free or voluntary caretakers for them. This would be the most important support for PLHA community.

Health service provider

Participants felt community support was needed for PLHA because of the stigma and discrimination associated with living with HIV. An online community would ensure privacy.

### Interface

Interface recommendations for an mHealth intervention included presenting content using pictures; using videos to present stories of role models; using push notifications for testing, appointment, and medication reminders; using geolocation to link to just-in-time (JIT) services; and using telemedicine to increase access to health care providers and community services.

Nearly all participants agreed that mHealth interventions should have minimal text if MSM and hijras were to use it regularly. They suggested presenting information in pictorial formats. Some participants’ expressed concerns about text-heavy content were potential user short attention spans and limited literacy, particularly among members of the hijra community.

Normally in our community, so many uneducated people are there and they cannot be able to know how this app would to be used.

Hijra

Health service providers agreed with these observations, noting that pictorial messages can aid in recall.

People generally understand the messages through pictures. When he sees the picture, he will try to recall that, “Yes, I have seen it somewhere.” So pictures should be used much.

Health service provider

Overreliance on written communication could impede understanding and utility of health messaging. Images or videos were a preferred method of messaging. Community participants expressed that an mHealth intervention could also explore areas beyond the traditional ambit of health education by using role models within the community and focusing on relationships with partners. An mHealth intervention could have role model stories from persons who have navigated stigmatizing environments to form successful social and romantic relationships with other MSM and hijras. An mHealth intervention could also provide role models of PLHA by describing the journey toward accepting one’s HIV positive status and living a happy and successful life.

[Include] videos on how people have become comfortable having HIV. Messages from role models. Some honest interview of what stages of approval [self-acceptance] the person has gone through... Something on lines of, “I was really angry, I was revengeful and I changed.”

MSM

Because of barriers to using SMS, push notifications when used selectively were an acceptable alternative. Because it was sometimes difficult to remember to test routinely or to remember other medical appointments, participants felt an mHealth intervention could be used to promote testing among community members by setting reminders. Reminders were perceived as being particularly useful for hijra sex workers as their working schedules often affect regular testing. For PLHA, push notifications were viewed as an acceptable method for reminders to take antiretroviral or other medications.

Participants were enthusiastic about the possibilities to access information on community events and JIT services. The most mentioned JIT services that the participants wished to see included were information on sexual and gender minority–serving nongovernmental organizations and testing centers, health care providers, counselors, legal aid, and helplines.

Some health service providers recommended telemedicine for mental health counseling and to broaden outreach, particularly to MSM and hijras who were closeted and hard to reach. One health care provider articulated the financial challenge to this approach while also highlighting the potential benefit.

Telemedicine broadens the outreach. [There is] considerable investment in the cost of equipment, [but] support would be counseling for mental health issues, since gay men don't find it easy to open up to everyone.

Health service provider

While health service providers and community participants acknowledged the infrastructure costs to begin offering telemedicine, the suggestions could allow an mHealth intervention to be accessed by a greater number of clients.

### Retention

Retention recommendations for an mHealth intervention included keeping it fun, developing content in regional languages, protecting confidentiality, and linking to social networking apps. Community participants insisted that the mHealth intervention be fun; otherwise, they felt members of the community would not access it regularly. They were wary of an intervention that was too serious because they felt that the members of the community would quickly lose interest in it. Another suggestion was to employ gaming mechanics. For example, 1 participant recommended employing a reward system for engaging in health-promoting behavior (ie, using a condom after last intercourse or getting tested for HIV).

There was a strong emphasis on use of local languages. English words could be misunderstood and would make the intervention inaccessible to members of the community who are less fluent in English. Both health service providers and members of the community expressed concern about the excessive use of English. For them, it was important that intervention developers embrace the multiculturalism of India.

Participants said protecting confidentiality on an mHealth intervention would be critical. Participants highlighted the stigma that the community faces and shared instances of how stigma affects uptake of services by the community. Participants stated that confidentiality would be a key aspect influencing use of an mHealth intervention by the community. Mindful that homosexuality is still illegal and highly stigmatized in India, participants said that if the intervention looked “too gay” or included nude content then it could potentially out users as sexual or gender minorities and discourage increased use of the intervention, as a substantial proportion of the community are closeted.

You should just have a logo and that’s about it. Logo or the identity interface should not have a gay feel. Even the name of the app can’t be too gay. And if there is a way that a person could hide the app, saying like you have this antivirus software where when it gets camouflage with something else.

MSM

MSM and hijras, in particular, were in favor of having access codes to ensure that the app remains private and confidential. However, they expressed concerns about information still being visible in instances of pop-up notifications. Participants recommended that confidentiality statements be featured prominently.

An additional recommendation to make the intervention more interactive and increase retention was to incorporate social networking. Participants described using various types of social media (eg, Facebook, Twitter) to improve reach. Some participants recommended that an mHealth intervention be linked to popular instant messaging apps (eg, Whatsapp) and dating apps (eg, PlanetRomeo) to allow messages to be delivered instantly by using instant message features on these apps for sending out health messages. By linking the intervention to preexisting social networking apps, an intervention could seamlessly be incorporated into participants’ lives rather than being another app they may forget to check.

## Discussion

### Principal Findings

Interventions using mHealth are becoming more feasible in India because of a burgeoning market for mobile technology and use of the Internet [18,19]. Although a demographic digital divide exists with millennials from higher income brackets being more likely to be mobile phone and Internet users [31], trends suggest a rapidly expanding technological market as products become more affordable. This creates new opportunities to develop culturally relevant mobile health (mHealth) interventions for a variety of health issues including HIV prevention.

Although many of the features identified by participants were similar to those reported in the literature using data from Western countries, there were some differences, including minimal use of text, the ability to deliver content in multiple regional languages, and concerns about confidentiality due to stigma and criminalization of homosexuality. For an mHealth intervention to be used by Indian MSM and hijra communities, it needs to feel safe, private, trustworthy, and confidential. To feel credible, it needs to include numerous safeguards to ensure confidentiality of the users and not appear “too gay.” An mHealth intervention could be an avenue for strengthening care and support services to MSM and hijra PLHA. Features such as reminders could improve adherence to medication and routine health care checkups. An intervention could also provide much needed emotional and physical support to PLHA. If the app mediated mental health counseling and access to JIT services and community events, it could empower members of the community to make informed choices about their health and sexuality.

### Strengths and Limitations

A strength of this study is the increased credibility resulting from triangulating data from focus groups with MSM and individual interviews with hijras, health service providers, and mHealth developers. However, data were collected only from persons living in Mumbai, so transferability to other Indian or South and Southeast Asian contexts, particularly to rural Indian contexts, is unknown. In addition, we were only able to recruit 4 hijras who had experience using technology to meet male sex partners. Similar to formative studies from Western countries, a limitation is that the focus was on preventive education, testing, and medication adherence rather than the on the entire continuum of care [27,32].

### Conclusion

Although participants recommended keeping the intervention simple, they also suggested a lot of content and features. When developing an intervention, it will be important for researchers, practitioners, and developers to remain cognizant of the trade-off between developing an intervention that will achieve the desired health outcome and addressing the diverse needs of potential users of the intervention. While members of the community and health service providers have some divergent opinions on content and retention features, there is broad support for the development of an mHealth intervention to prevent HIV transmission and support persons living with the virus. Where differing views exist, such as use of pictures of graphic STIs, additional research is needed to ensure uptake of messaging. However, it should be noted that fear-based messaging techniques for HIV prevention have been shown to be ineffective [33]. Although our findings do not represent a framework for a particular intervention, practitioners and researchers developing interventions for Indian MSM and hijras should consider the wishes of the community if they decide to incorporate mobile technology into their prevention efforts.

## Abbreviations

IQR: interquartile range
JIT: just-in-time
MSM: men who have sex with men
PLHA: persons living with HIV/AIDS
SMS: short message service
STI: sexually transmitted infection
